# Supplementary material for: Trend analysis and prediction of fabric tear performance testing processes based on the BLTT-FT model
Source: PLoS One. 2025 Dec 1;20(12):e0336501. doi: 10.1371/journal.pone.0336501 (PMC12668543; doi:10.1371/journal.pone.0336501)
Supplement: Attachment — Submitted filename: Response to Reviewers.pdf [file pone.0336501.s001.pdf]

Response to reviewer, PONE-D-24-30488

Dear Editors and Reviewers,

We are grateful for the opportunity to revise our manuscript entitled " **A Data-Driven Study of Predictive Analysis of Fabric Tear Experiments**". Thank you very much for your comments and professional advice. These opinions help to improve academic rigor of our article. Based on your suggestion and request, we have made corrected modifications on the revised manuscript. Meanwhile, the manuscript was also professionally reviewed. We hope that our work can be improved again. Furthermore, we would like to show the details as follows:

Additional Editor Comments

1.Provide more comprehensive details about the datasets, including the total size, characteristics, and sources of the fabric elasticity grades. Clarify whether the datasets represent real-world scenarios and ensure diversity in fabric types for broader applicability.

Response:

Thank you for your question, I have explained in detail in the body of the thesis to address this issue you raised:

① In Table 2 Three types of data sets in Chapter 4, the number of samples has been added. In order to increase the generalization of the experiment, there are 150, 170 and 160 types of fabrics with high, medium and low elasticity, respectively (I would like to apologize to you for the mistake in filling in the number due to my previous miscalculation, which has been changed now). A total of ten experiments were conducted for each type in both warp and weft directions, basically including most fabric fabrics, ensuring a diversity of fabric types for wider applicability.

② The fabrics used in the experiments were all samples sent to the third-party testing laboratory by various textile companies, and the data sources were formal and very representative of real-world scenarios.

③ For the size of the fabrics, Chapter 3 has made detailed sampling specifications, in strict accordance with international standards: “ISO 139372-2000 Textile fabrics - Tear properties - Part 2: Determination of tear strength of trouser specimens”.

2. Elaborate on the specific contributions of each component in the Bi-LSTM-Trans-TCN architecture. Explain why a combination of Bi-LSTM, Transformer, and TCN is ideal for this task compared to simpler or alternative architectures.

Response:

Thank you for your question, and I have explained in detail in the body of the paper in response to your question:

The Bi-LSTM-Trans-TCN architecture proposed in this paper is based on the Transformer model, which is modified by introducing the BiLSTM model consisting of Time Convolutional Networks (TCNs) and improved LSTMs, which is developed in detail in Chapter III. The reason for not using simple or alternative architectures is because its three can have some instability when used independently, resulting in less accurate prediction results. After consulting the latest authoritative literature in the field of prediction, it was found that the simple splicing of prediction models was still not very accurate and could not meet the requirement of high accuracy prediction in this experiment, so after many experiments and adjustments, the Bi-LSTM-Trans-TCN architecture was built to realize the requirement of high accuracy prediction.

Firstly, the Transformer model is used as the base model. Since the Transformer model is set for machine translation, it can not be directly used as the prediction time series, so its encoder part is used as the basic model. Then Temporal Convolutional Network (TCN) was introduced to modify it to make it more suitable for the prediction of time-series data in this paper, TCN can capture both high-level and low-level features with stable gradient and make the model able to process the time-series information in parallel, which improves the prediction accuracy of the model and the training efficiency. The improved Transformer model can take full advantage of Transformer and TCN for better prediction of sequence data, but its network structure lacks sequential information, and although its positional encoding uses sine and cosine to model positions, it is not sufficient for complete modeling and there is some lack of information. Therefore, before transmitting the data to its multiple self-attention points, it will be processed using a bidirectional long and short-term memory network to capture the sequential dependency, and finally, due to the large sample size of the dataset, a BiLSTM network model consisting of a modified LSTM is also used to speed up the data learning. Thus summarizing, the Bi-LSTM-Trans-TCN prediction architecture in this paper i.e., it has an advantage in accuracy and also in speed.

3. The paper lacks comparisons with existing models or techniques. Include performance benchmarks against well-known methods like traditional LSTM, GRU, or TCN-only models to substantiate the claimed superiority of the proposed approach.

Response:

Thank you for your question, and I have explained in detail in the body of the paper to address this issue you raised:

In order to verify the effectiveness of the Bi- LSTM-Trans-TCN model in monitoring the power change during the fabric tearing performance monitoring experiments, five common prediction models are selected for comparative analysis in Chapter 5 of this paper, which include the LSTM model, the BiLSTM model, the Transformer model, the TCN model, and the Transformer- LSTM model for prediction performance comparison. These models are the original models used to construct the models in this paper as well as the combined models studied in existing authoritative articles, and it is more convincing to verify the advantages of the proposed models in this paper by comparing them with these models.

By comparing the loss function decline curves of the training set and the test set during the training of different models on the dataset, it is verified that the loss curve of the Bi-LSTM-Trans-TCN architecture proposed in this paper not only declines more, but also has a shorter fitting time, and the loss curves of the test set are closer to the training set, which makes the training effect better. Moreover, it is obtained through multi-step prediction that the model proposed in this paper has excellent performance and strong generalization ability in the prediction of electric power parameters during fabric tearing experiments with different elasticity levels.

4. While MAE and MAPE are reported, introduce additional metrics such as RMSE, R2, or F1 score for a more evaluation of prediction accuracy and reliability.

Response:

Thank you for your question, I have explained in detail in the body of the thesis to address this issue you raised:

In the table of multi-step prediction evaluation metrics for the three datasets in Chapter 5, not only MAE, RMSE, MAPE, and MAE I also added R^2 evaluation metrics to better assess the accuracy and reliability of the predictions. In datasets 1, 2, and 3, the average R^2 for 1-step

prediction is as high as 0.9865; for 4-step prediction it is as high as 0.9600; and for 9-step prediction it is as high as 0.9252.

5. Discuss the computational complexity of the proposed model and its scalability for large datasets. Include runtime analysis or resource requirements, especially for real-time predictions.

Response:

Thank you for your question, and I have explained in detail in the body of the thesis in response to this issue you raised:

In the model training and parameter setting in Chapter 4, it is proposed that the experimental system of this research runs on Windows 11 operating system with Intel(R) Core(TM) i5-8250U CPU @ 1.60GHz processor, 16.0GB of system RAM, Python version 3.9 installed in the experimental environment, Pytorch version 1.13.1.

And the dataset data volume is particularly large so the offline training method is used, each dataset is divided into long, medium and short data segments, and each dataset is inputted into the Bi-LSTM-Trans-TCN model in parallel for offline training. In the online application, when the first set of electric power data of the experimental process is obtained, the state window will scroll to the next state point and the model will be updated every time the "change-stretch-reset" operation is completed, and the incremental learning will be repeated to update the database, forming a closed loop of continuous iteration and optimization. The model is used to predict the next electric power data with appropriate training parameters, and the results show that the parameter settings in this paper can smoothly carry out the prediction experiments and reduce the complexity of the model operation.

6. Incorporate detailed visualizations, such as prediction vs. actual trends, error distribution plots, and model attention maps, to enhance the clarity of results and model interpretation.

Response:

Thank you for your question, and I have explained in detail in the body of the thesis to address this issue you raised:

I added the multi-step prediction error visualization plots of the data set (1-3) in Chapter 5 to make it more convenient to observe the changes of the evaluation indexes of multi-step prediction

of different elastic fabrics in the experimental process on the basis of the table, and made a detailed write-up in text to enhance the clarity of the results and model interpretation.

The smaller the values of MAE, MSE, RMSE and MAPE, and the closer the R^2 is to 1, the smaller the error between predicted and actual power is, indicating the better prediction performance of the model. It can be seen that the Bi-LSTM-Trans-TCN model in this paper has excellent prediction effect in the three data sets and stable prediction effect in different data sets. Moreover, the trend graphs of the three datasets in one step, four steps and nine steps are plotted by comparing the predicted values with the real values, and the comparison graphs of the predicted values with the real values also show the excellent prediction effect of the model, and the results of the proposed method based on Bi-LSTM-Trans-TCN are compared with the predicted results of other prediction models in the steps of 1, 4 and 9 steps there is still a high degree of fit between the actual and predicted curves.

7. Assess the model's ability to generalize across unseen datasets. Conduct experiments with cross-validation or on completely different types of fabrics to demonstrate its robustness.

Response:

Thank you for your question, and I have explained in detail in the body of the paper to address this issue you raised:

In order to increase the generalization of the model, I did experiments on high, medium and low elasticity fabrics, and the number of fabrics of each elasticity is large, and the experimental results show that the Bi-LSTM-Trans-TCN based method has excellent performance and strong generalization ability in predicting the electric power parameters during the experimental process of tearing fabrics with different elasticity levels.

8. Expand on how the proposed method could impact industrial fabric testing practices. Acknowledge limitations, such as sensitivity to noisy data, and suggest potential improvements or future work to address these challenges.

Response:

Thank you for your question, and I have explained in detail in the body of the thesis in response to this issue you raised:

① This study originates from the demand for real-time monitoring and optimization of fabric tearing experiments in the field of textile production and quality control, and for the limitations of the traditional means of monitoring the experimental process, we propose a research on the application of Bi-LSTM-Trans-TCN model based on electric power sequences in the prediction of fabric tearing experimental process. Based on the multi-source data-driven situational awareness system for fabric tearing performance detection, we have improved and perfected the system function of situational awareness, and added the overall prediction function of the fabric tearing experimental process, with a view to improving the efficiency and accuracy of the experiments, as well as reducing the experimental processes and optimizing the whole experimental process to a certain extent. The prediction model proposed in this paper can well predict the next 1 group, 4 groups or even 9 groups by the experimental data of tearing of a tissue, even when the prediction range is very large, it can also achieve good prediction effect, which can make it possible to get other or even 9 groups of experimental data in the actual experiments by only one group of experiments of the torn experimental fabric. To a certain extent, process optimization has been carried out to improve the experimental efficiency and make the fabric tearing experiment further develop towards intelligence.

② Limitations: We plan to deepen the optimization of the model and further enhance the prediction accuracy and generalization ability of the model by integrating more types of sensor data, such as temperature, humidity and other environmental parameters. Because the physical properties of fabrics are easily affected by temperature and humidity during the experiment, it is necessary to include environmental influences in future research to reduce the uncertainty brought by external factors to the experiment.

Improvement program for noise data sensitivity. In this paper, during the data pre-processing, only the noise signal of the sensor is smoothed, and more advanced signal filtering technology should be used in the subsequent research, and we plan to introduce the adaptive filtering algorithm, which is able to dynamically adjust the filter parameters according to the real-time characteristics of the noise signal, so as to remove the noise interference more effectively. At the same time, in the data acquisition stage, increase the signal shielding measures to reduce the impact of electromagnetic interference, through these improvements, it is expected that the noise-induced test deviation can be reduced to within 5%.

Thank you very much for your attention and time. Look forward to hearing from you.

Yours sincerely,

Yang Lu

December 15, 2024

Zhejiang Light Industrial Products Inspection and Research Institute

Zhejiang University of Science and Technology.

Hangzhou 310000,China.

E-mail: 13857154948@163.com
