# Supplementary material for: Trend analysis and prediction of fabric tear performance testing processes based on the BLTT-FT model
Source: PLoS One. 2025 Dec 1;20(12):e0336501. doi: 10.1371/journal.pone.0336501 (PMC12668543; doi:10.1371/journal.pone.0336501)
Supplement: Attachment — Submitted filename: Response_to_Reviewers_auresp_2.pdf [file pone.0336501.s002.pdf]

Response to reviewer, PONE-D-24-30488

Dear Editors and Reviewers,

We are grateful for the opportunity to revise our manuscript. Thank you very much for your comments and professional advice. These opinions help to improve academic rigor of our article. Based on your suggestion and request, we have made corrected modifications on the revised manuscript. Meanwhile, the manuscript was also professionally reviewed. We hope that our work can be improved again. Furthermore, we would like to show the details as follows:

Reviewer #1:

You have incorporated all the suggestions. Its good now, but still the paper needs proofreading in detail.

Response:

Thank you for your valuable suggestions! We have systematically proofread the full paper in strict compliance with academic standards and journal requirements: we not only checked the formatting details against the journal's Submission Guidelines one by one, but also cross-validated the logic of the research methodology, the accuracy of the data citation and the rigor of the conceptual presentation to ensure that the content complies with the standards of academic publishing. The full text has now passed the professional proofreading process. Thank you again for your careful guidance on the quality of the paper.

Reviewer #2:

This paper presents a data-driven method for real-time prediction of fabric tearing experiments using a Bi-LSTM-Trans-TCN model based on electrical power sequences. By integrating bidirectional temporal dependencies, global sequence attention, and temporal convolutional efficiency, the model achieves high multi-step prediction accuracy across fabrics of varying elasticity. The approach offers promising implications for enhancing experimental efficiency and process optimization in textile durability testing. This is an interesting paper, but several issues still need to be addressed:

Response:

Considering that the name of the original model is too long, and since this study adopts a trend prediction model for fabric tearing performance detection experimental processes based on a "bidirectional long-short-term attention mechanism", the model is named "BLTT-FT".

1.*The paper has six co-authors; therefore, the "Author Summary" section should use "We" instead of "I" to reflect collective authorship.*

Response:

Thank you for pointing out this issue! We fully acknowledge the collective nature of authorship and have revised the "Author Summary" section to consistently use "We" throughout, ensuring it accurately reflects the collaborative efforts of all six co-authors.

2.*The statement “The Transformer model is set up for machine translation and cannot be used directly as a predictive time series” is inaccurate and should be revised. Numerous studies have directly applied Transformer architectures to time series prediction and classification tasks.*

Response:

Thank you for pointing this out! We have made the change and changed the relevant text inside the “Trans-TCN network construction” section of the text to read: “Although the Transformer model was originally designed for machine translation, it can still be applied to time series prediction by exploiting its architectural potential. Therefore, its encoder part is used as the basic model.”.

3.*When introducing parameters in equations, "where" should be aligned to the left and begin with a lowercase letter.*

Response:

Thank you for pointing this out! We have adjusted the equations so that "where" is left-aligned and starts with a lowercase letter, following the specified formatting guidelines.

4.*The "Contributions" section should focus on the theoretical or practical advancements of the work rather than repeating a summary. For example, the third listed contribution should be deleted. The authors are encouraged to revise this section with reference to recent works in deep learning-based fault prognosis, such as Reliability Engineering & System Safety (DOI: 10.1016/j.ress.2024.110014).*

Response:

Thank you very much for your valuable suggestions! We fully agree with your guidance that the “Contributions” section should focus on theoretical or practical innovations. Based on your suggestion, we have reorganized and rewritten this section to remove redundant summaries and highlight the innovative breakthroughs and practical applications of our research in the field of deep learning fault prediction. At the same time, we have carefully read the paper and added citations to the literature in the chapter of “Predictive applications of time series data” (Ref. 31), in order to enhance the relevance of this paper to the cutting-edge research in the field and the depth of argumentation.

5.*If the experimental data were collected by the authors themselves, this is a notable strength. However, in that case, the description of the experimental process and dataset characteristics must be significantly expanded.*

Response:

Thank you for pointing out this critical issue! As you mentioned, the experimental data were collected independently by our team, which is indeed one of the core strengths of this study. In order to fully reflect this advantage, we have systematically optimized the experimental part: on the one hand, we have added the analysis of significance experiments to verify the reliability of the results by statistical methods; meanwhile, we have supplemented the ablation experiments to deeply analyze the contribution of each module to the final results, in order to enhance the logic of the argumentation. On the other hand, in the section of “Data preparation and processing”, the collection process, sample distribution, data pre-processing methods, key feature indicators, etc., are elaborated in detail, and new data visualization charts are added to assist illustration, to ensure that the features of the dataset are presented comprehensively and clearly. This revision has further strengthened the research foundation of the study.

6.*The paper should clearly distinguish between ablation and comparative experiments. Additionally, more recent models should be included in the comparative experiments to better demonstrate the superiority of the proposed method.*

Response:

Thank you for your suggestion! We have added the ablation experiment in the paper, which shows that the multi-module combination effectively solves the problems of detail accuracy of single-step prediction and long-range dependence of multi-step prediction through the hierarchical modeling of bidirectional time-series capturing, global attention correlation, and local feature extraction, and the synergistic effect is especially significant in multi-step prediction scenarios, which provides a better solution for multi-scale prediction of time-series data. This study focuses on the innovation of intelligent prediction methods in textile manufacturing scenarios, and traditional models such as LSTM and Transformer are chosen for comparison experiments because they are the mainstream solutions in the field of textile engineering, and the ablation experiments have already verified the necessity of hybrid architectures through component disassembly. The existing comparisons form a complete logical chain, which is sufficient to support the core conclusions. As for the recent emerging time-series architectures, there is a contradiction between their reliance on large-scale data and the sample size limitation of textile experiments, and this study, as the development of customized algorithms in the field, is not included in the scope of the comparison for the time being, and the related explorations will be used as the direction of future work.

Additional Editor Comments

1.*Methodological rationality issues: Although the motivation for combining Bi-LSTM, Transformer, and TCN is understandable, the rationale is only described qualitatively, lacking component ablation studies on the contributions of each module (BiLSTM, Transformer, TCN).*

Response:

Thank you for your suggestion! We have added ablation experiments in the paper. The ablation experiments show that the combination of multiple modules effectively addresses the issues of single-step prediction detail accuracy and long-range dependency in multi-step prediction through hierarchical modeling of bidirectional temporal sequence capture, global attention correlation, and local feature extraction. Their synergistic effect is particularly significant in multi-step prediction scenarios, providing a better solution for multi-scale prediction of time-series data.

2. *Model structure explanation issues: The structural differences between the improved LSTM and the standard LSTM are not clearly explained, nor are the impacts of such differences on performance or complexity.*

Response:

Thank you for your suggestion! We have provided a detailed explanation in the section "Bi-LSTM Network Construction": Due to the large sample size of the dataset, the traditional LSTM, although performing well, leads to a large number of parameters that need to be learned for each gate, which results in a long-running time for the LSTM, so an improved LSTM network is proposed to speed up the data learning.

3. *Chart issues: Although Figures 2-4 are helpful, the information is overly dense, and annotations and layer specification instructions need to be added.*

Response:

Thank you for your suggestion! Figures 3-4 show the traditional algorithm framework, and the corresponding annotations have been noted in the text. Figure 2 illustrates the "overall network structure of the BLTT-FT algorithm" in my thesis, with the corresponding annotations explained in the text.

4.*Comparative experiment issues: The paper only compares with several traditional deep learning models (such as LSTM, Transformer, etc.), lacking many recent architectures that have shown excellent performance in time-series modeling.*

Response:

This study focuses on innovations in intelligent prediction methods for textile manufacturing scenarios. Traditional models such as LSTM and Transformer were selected for comparative experiments because they are mainstream solutions in the field of textile engineering, and ablation experiments have validated the necessity of the hybrid architecture through component decomposition. The existing comparisons form a complete logical chain, sufficiently supporting the core conclusions. As for recent emerging time-series architectures, their dependence on ultra-large-scale data contradicts the limitations of textile experiment sample sizes. As a domain-specific algorithm development, this study does not currently include them in the comparison scope, and related explorations will be considered as future work directions.

5.*Evaluation metric issues: Evaluation metrics for all methods and datasets should be standardized (e.g., MAE, RMSE, R^2, MAPE), as the current comparisons are fragmented.*

Response:

Thank you for your suggestion! We have added "significance test analysis" to the paper, which explains "A one-way ANOVA was conducted to test the MAE, RMSE and MAPE indicators of different samples under each prediction step, and the results showed that there were extremely significant differences among different samples under all indicators and prediction steps.”

6.*Issue of insufficient theoretical discussion: There is minimal discussion on time complexity, scalability, and convergence stability.*

Response:

Thank you for your suggestion. This study has fully validated the convergence stability of the model through loss function curves, architectural design mechanisms, and comparative experiments. The loss curves of the training and test sets for the model continuously and smoothly decline within 100 epochs, with a difference always less than 0.002, and the test set loss does not exhibit overfitting-induced increases, demonstrating a stable convergence process.

7. *Issue of missing model setup details: The performance of the model under online learning and offline batch settings is unclear, and more detailed information on training time, hardware, and memory requirements is needed to ensure reproducibility.*

Response:

Thank you for your suggestion. In the chapter "Model training and parameter setting", the issues you mentioned are elaborated in detail, and the data are presented in the form of tables.

8. *Chart supplementation issues: Although some figures are included, the paper still needs trend charts of predicted values versus true values (for different step sizes) and error distribution plots to support the claims of interpretability.*

Response:

Thank you for your suggestion. We have supplemented this in the paper. Figure 14 shows the error visualization diagram, and Figures 15, 16, and 17 show the trend diagrams of predicted values versus true values (for different step sizes).

9.*Issue of insufficient generalization evidence: The authors claim that the model has strong generalization ability, but this is only based on three internal datasets, lacking external or cross-domain validation.*

Response:

The three datasets constructed in this study are not simple repetitions of similar samples but cover the variation range of fabric elasticity grade, a core physical property (see Table 2). In the field of textile materials, elasticity is a key factor determining the tearing performance of fabrics, and the breadth of its value range directly reflects the model's adaptability to mainstream working conditions in actual production scenarios. Therefore, the model's excellent performance on three typical datasets of low-elasticity, medium-elasticity, and high-elasticity fully demonstrates its internal generalization ability for the target task—that is, it can effectively adapt to the prediction needs of different material properties within the same production system. This aligns with the conventional evaluation logic of "validating model robustness based on material property gradients" in textile industry quality control.

Regarding "external or cross-domain validation," this study focuses on model optimization and system integration within a single scenario, with the validation scope limited to multi-working-condition data in the same laboratory environment. If "cross-domain" is defined as production data across enterprises or devices, its validation would involve collaborative adjustments of different hardware configurations and process parameters, which belongs to an extended research direction beyond the core scope of this study. Nevertheless, we acknowledge the importance of cross-domain generalization and plan to collaborate with industry partners in follow-up work to conduct transfer learning validation based on production data from different textile enterprises, further improving the model's engineering applicability.

10. *Practical deployment-related issues: The robustness in the presence of noise, class imbalance, or data missing is not discussed. Additionally, there is an overstatement of industrial relevance, with a lack of actual deployment or pilot demonstrations.*

Response:

This study focuses on model architecture innovation and laboratory environment validation, aiming to provide a reusable algorithm prototype for industrial applications. Issues such as edge computing adaptation and robustness under extreme working conditions that need to be further addressed in actual deployment belong to the category of subsequent engineering iterations. Currently, the team has reached an agreement with a collaborative textile laboratory to conduct experiments in noisy data scenarios on its semi-industrial test line, and the relevant validation results will be included as extended content in future research.

The discussion of industrial relevance in this study is based on real needs (such as reducing experimental procedures and improving efficiency), rather than exaggeration. This conclusion is drawn from real experimental processes and data, with no overstatement.

11.Language issues: English grammar and syntax need to be edited to improve clarity.

Response:

Thank you for pointing out the language issues. We have carefully revised the English grammar and syntax throughout the manuscript to improve clarity, and the language has been polished to ensure accuracy and

smoothness. All related issues have been addressed, and we believe the revised version now meets the language requirements of the journal.

12.*Information update issue: The email addresses and author information need to be updated according to the requirements in the cover letter.*

Response:

The email information has been updated in the system, but the modified email still seems unable to receive correspondence from your journal.

13.*Title issue: The title should be more specific, for example, "...using Bi-LSTM-Trans-TCN based on Electrical Power Sequences".*

Response:

It has been revised to:Trend Analysis and Prediction of Fabric Tear Performance Testing Processes Based on the BLTT-FT Model.

Considering that the name of the original model is too long, and since this study adopts a trend prediction model for fabric tearing performance detection experimental processes based on a "bidirectional long-short-term attention mechanism", the model is named "BLTT-FT".

Considering the large number of diagrams in the paper and the fact that the contents of the diagrams are closely related to the core arguments of the paper, the reasonableness of the diagram layout is crucial for the readers to understand the research results. In order to ensure the accuracy and completeness of the information conveyed, **Figure 4, Figure 8, Figure 9, Figure 13, Figure 14, Figure 15, Figure 16 and Figure 17** in the thesis are attached as the reference examples of layout. These examples have been repeatedly optimized in terms of chart layout, labeling standard and echoing with the main text, which can effectively improve the presentation of information. I urge you to refer to them in the layout of your magazine, and if there is any need for additional explanation, I am ready to cooperate with the communication. Thank you for your attention and support of this paper!

Thank you very much for your attention and time. Look forward to hearing from you.

Yours sincerely,

Yang Lu

May 29, 2025

Zhejiang Light Industrial Products Inspection and Research Institute

Zhejiang University of Science and Technology.

Hangzhou 310000,China.

E-mail: 13857154948@163.com
