# Supplementary material for: Trend analysis and prediction of fabric tear performance testing processes based on the BLTT-FT model
Source: PLoS One. 2025 Dec 1;20(12):e0336501. doi: 10.1371/journal.pone.0336501 (PMC12668543; doi:10.1371/journal.pone.0336501)
Supplement: Attachment — Submitted filename: report.pdf [file pone.0336501.s003.pdf]

Here is a comprehensive academic review of the manuscript titled "**Trend Analysis and Prediction of Fabric Tear Performance Testing Processes Based on the BLTT-FT Model**", with a section-by-section evaluation. The overall recommendation is Minor Revision, as the work presents significant methodological contributions and empirical rigor, but some areas still require refinement.

1. Abstract and Author Summary

The abstract clearly outlines the research objective, methodology, and key results. It effectively communicates the novelty of the BLTT-FT model and emphasizes its predictive capabilities in multi-step forecasting of fabric tearing performance. The inclusion of quantitative metrics (e.g., RMSE = 0.0881, MAE = 0.0609, MAPE = 3.06%, R^2 = 0.9572) strengthens the abstract's credibility. However, the abstract could benefit from a more concise articulation of the model's architecture and clearer distinction between data-driven methodology and engineering application.

The author summary is well-structured and now uses the proper collective pronoun ("we") as per the reviewers' comments. It provides a helpful bridge between technical content and applied context. The emphasis on industry relevance and experimental efficiency is commendable, though the summary could more clearly define how the model integrates with existing textile manufacturing systems.

2. Introduction

The introduction successfully contextualizes the significance of fabric tear testing in the textile industry, particularly for elastic fabrics. It provides a solid literature background and justification for shifting from traditional to intelligent monitoring methods. The discussion on indirect monitoring via electrical power sequences is novel and well-motivated. However, the section could be improved by more explicitly stating the research gap in current machine-learning-based predictive models for time-series in this domain. Also, while the "change–tensile–reset" cycle is critical to the study, its definition appears too late and could be introduced earlier to ground the reader.

3. Related Work

This section is thorough and reflects an extensive review of the state-of-the-art in AI-based fabric performance prediction and time-series analysis. The authors effectively position their work relative to ARIMA, BiLSTM, Transformer, and TCN models. The citation of recent literature—including works published in 2023 and 2024—is a strength. However, the review of time-series forecasting could be further enriched by discussing limitations of existing models specifically in small-sample industrial setups, which is the context of this study. The integration of [31] as a reference to fault prognosis is appropriate and aligns with reviewer suggestions.

4. Model Architecture (BLTT-FT System Network Construction)

The proposed hybrid model—comprising improved BiLSTM, Transformer encoder, and TCN layers—is well-justified. The architectural novelty lies in the hierarchical fusion of temporal dependencies (short-term and long-term) and local feature extraction. The description is

technically sound, and the equations are clearly presented. The use of modified LSTM with reduced gates is a noteworthy engineering contribution to mitigate computational overhead.

However, the section would benefit from clearer comparative commentary on the trade-offs in complexity, training time, and accuracy between the standard and improved versions of LSTM and Transformer. Additionally, while the visual diagrams (e.g., Fig. 2–4) are helpful, they remain dense, and further annotation or layer-wise explanation in the captions would improve interpretability.

5. Data Preparation and Processing

This section is particularly strong in its attention to the experimental protocol, which follows ISO standards. The authors' emphasis on real-time data collection using electrical power signals is innovative. The decision to use only power data—due to its robust correlation with mechanical characteristics—is well-supported.

The preprocessing steps (LOCF imputation, smoothing, and anti-leakage procedures) are standard and appropriate. The event-driven rolling window segmentation is a major methodological contribution and is well-explained. However, the rationale for choosing specific window lengths or prediction steps (1, 4, 9) could be better justified, perhaps through a sensitivity analysis.

6. Experimental Setup and Parameter Settings

The training details—hardware configuration, environment, and hyperparameter tuning—are clearly presented and reproducible. The use of three datasets with varying elasticity properties (low, medium, high) enhances the generalizability of the model. The offline training time (8 hours) and online adaptation time (200 ms) are reasonable for industrial applications.

The inclusion of both long and short data segments in model training (long = 10 specimens, short = 1) is a robust design choice. However, the paper lacks a discussion on class imbalance (if any) across fabric types, which could affect model performance.

7. Comparative and Ablation Experiments

The comparative experiments are rigorously conducted across five baseline models (LSTM, BiLSTM, Transformer, TCN, Transformer-LSTM). The BLTT-FT model consistently outperforms baselines, especially in 9-step prediction, where it achieves MAE = 0.0762 and R^2 = 0.9211 on dataset 1. The degradation in performance with increasing step size is natural but remains significantly better than other models.

The ablation study is a major strength and clearly demonstrates the importance of each module (BiLSTM, Transformer, TCN). For instance, removing the Transformer results in a 57% increase in MAE for 9-step prediction. This validates the synergistic design of the full model.

However, while the inclusion of ablation experiments addresses prior reviewer concerns, the naming conventions (Model 1–7) could be more intuitive if consistently used across tables and text. Also, the comparative tables could benefit from statistical significance indicators (e.g., asterisks on best values).

8. Significance and Variance Analysis

The use of ANOVA to statistically validate the differences in prediction error (MAE, RMSE, MAPE) across datasets and prediction steps is commendable and addresses a critical reviewer suggestion. The F-values and p-values are clearly reported, confirming that performance differences are not due to random variation.

The inclusion of box-and-whisker plots (Figs. 18–20) offers a visual summary of error distribution, but figure captions could better highlight key insights (e.g., dataset 2's superior MAPE stability). Additionally, although the significance testing is well-executed, a post-hoc test (e.g., Tukey HSD) could be suggested as future work to determine which datasets differ significantly from each other.

9. Conclusion and Prospects

The conclusion effectively synthesizes the contributions and empirical findings. The summary of performance metrics across all datasets (e.g., average RMSE = 0.0881, MAE = 0.0609, R^2 = 0.9572) reinforces the model's robustness. The claim that the model can predict 9 future operations using only the first is strongly supported by results.

The future work section is realistic and well-structured. The authors acknowledge limitations such as the need for environmental sensor integration, better noise filtering, and cross-domain validation. These acknowledgments reflect a mature understanding of the model's current scope and scalability.

Strengths:

Novel hybrid model architecture combining BiLSTM, Transformer, and TCN.

Empirical rigor across multiple datasets with strong generalization.

Effective response to reviewer feedback, especially regarding ablation studies and statistical validation.

Clear industrial relevance and potential for real-time application.

Areas for Improvement:

Enhance figure clarity and add annotations.

Justify window size and prediction steps with empirical or theoretical rationale.

Discuss class imbalance or dataset distribution properties.

Add commentary on model interpretability and limitations for deployment.

Recommendation:

Minor Revision — with targeted improvements to clarity, figure presentation, and technical justifications.
