# Supplementary material for: Trend analysis and prediction of fabric tear performance testing processes based on the BLTT-FT model
Source: PLoS One. 2025 Dec 1;20(12):e0336501. doi: 10.1371/journal.pone.0336501 (PMC12668543; doi:10.1371/journal.pone.0336501)
Supplement: Attachment — Submitted filename: Response_to_Reviewers_auresp_3.pdf [file pone.0336501.s004.pdf]

Reviewer #2: The manuscript has been significantly improved through the revisions. The key issues raised in earlier rounds have been addressed adequately, and the current version demonstrates a clear structure, enhanced clarity, and stronger experimental support. From an academic standpoint, the paper is now suitable for consideration for acceptance.

Answer：

Thank you very much for your recognition and careful review of the manuscript! Your affirmation is an important motivation for us to continuously improve our research.

We would like to thank you for the key questions you raised in the previous rounds of review, which pointed out the direction of revision and helped us to further organize the structure of the paper, improve the clarity of the content, and strengthen the rigor of the experimental support. Your professional guidance is indispensable for the improvement of the current version.

We will continue to maintain a rigorous academic attitude, and will be ready to listen to your suggestions if there is any further improvement needed. Thank you again for your hard work and support of this study!

Reviewer #3: A prediction model that combines the improved BiLSTM structure, the Transformer coding layer and the TCN network layer is presented in the work. Some revisions are needed.

1. *The short-term attention mechanism is used in this article. Why did the author use this attention mechanism instead of other attention mechanisms? Is there a theoretical necessity?*

Answer：

Thank you for your suggestions!

The short-term attention mechanism used in this paper (mainly reflected in the synergy between the improved BiLSTM and TCN modules) was chosen based on the inherent characteristics of the electric power sequence in the fabric tearing experiments and the needs of the multistep prediction task, with a clear theoretical necessity, which is illustrated in the following three aspects in conjunction with the research content:

①Short-term dynamics of the adapted electric power sequence

The electric power sequence in the fabric tearing experiments is characterized by significant short-term fluctuations. There are significant short-term fluctuations in the sequence: in the core stretching phase, the yarn breaks in sequence, triggering instantaneous changes in power (e.g., peak fluctuations); the power changes in the sampling and reset phases are relatively smooth, but still show local temporal correlations (e.g., the gradual change of power with the motion of the fixture in the reset phase). The time scales of these features are mostly milliseconds to seconds, which are typical short-term dynamic patterns that directly reflect the real-time status of the experimental process.

The design of short-term attention mechanisms can precisely target such features: the improved BiLSTM can accurately extract bi-directional short-term temporal correlations (e.g., before and after the sudden change of power in the stretching phase) by simplifying the gating structure (only 1 output gate is retained), and the expanded causal convolution of the

TCN can avoid the loss of short-term details by focusing on sequence segments through the local perceptual field, and by combining with the The expansion causal convolution of TCN focuses on sequence segments through the local perceptual field, together with the residual connection to avoid the loss of short-term details, and can effectively capture the local power change rule in the stages of sample change and reset.

② Make up for the inherent limitations of the long-term attention mechanism The self-attention mechanism of the Transformer coding layer in the

model is responsible for modeling global information (e.g., the difference in the overall power trend of different elastic fabrics), but there is a natural defect in dealing with the short-term details: its global computation smoothes out the local dramatic fluctuations, and it is susceptible to the interference of high-frequency noises (e.g., small power fluctuations induced by the vibration of the equipment), which leads to a decrease in the accuracy of short-term prediction. Accuracy degradation.

The short-term attention mechanism solves this problem by complementing the long-term mechanism:

BiLSTM's bi-directional temporal sensing can strengthen the local contextual association, avoiding the Transformer's "over-smoothing" of short-term mutations;

TCN's local convolution and weight normalization can inhibit the high-frequency noise, ensuring the stability of short-term feature extraction (smoothing in preprocessing). stability of short-term feature extraction (the smoothing process in preprocessing further assists this process).

③Ablation experiments to verify the necessity

The results of the ablation experiments in the paper directly prove the irreplaceability of the short-term attention mechanism:

After removing the BiLSTM module, the 1-step predicted MAE of Dataset 1 increases from 0.0489 to 0.0984 (up 50.3%), which indicates that its ability of bi-directional short-term temporal modeling is crucial for capturing the instantaneous dependency;

After removing the TCN module, the 4-step predicted MAE of Dataset 2 increases from 0.0984 to 0.0489; After removing the TCN module, the 4-step predicted After removing the TCN module, the 4-step prediction RMSE of Dataset 2 increases from 0.0931 to 0.2671 (up 68.8%), which confirms the central role of its localized feature extraction in suppressing the accumulation of short-term multi-step prediction errors.

In summary, the choice of short-term attention mechanism is not a subjective preference, but an inevitable result based on the characteristics of experimental data (short-term dynamics is significant), the need for model complementarity (to make up for the defects of the global mechanism) and experimental validation (to dissolve the experimental support), forming a synergistic framework of"local details-global trend"with the long-term attention mechanism (Transformer), and finally realizing the synergistic framework of the fabric prediction. The synergistic framework finally realizes the high-precision real-time prediction of the fabric tearing experimental process.

2. Improved BiLSTM structure, the Transformer coding layer and the TCN network are integrated. What is the theoretical reason for this integration?

Answer:

The theoretical reason for integrating the improved BiLSTM structure, the Transformer coding layer and the TCN network stems from the contradiction between the complex timing characteristics (short-term fluctuations, long-term dependence, variable length) of the electric power sequence in fabric tearing experiments and the limitations of a single model, which form a synergistic framework by complementing each other's functions, and can be analyzed from the following perspectives:

① Improved BiLSTM: Addressing the need for modeling bidirectional short-term timing dependence : Addressing the need for modeling bi-directional short-term temporal dependence

There are significant short-term dynamic correlations in the electric power sequences of fabric tearing experiments (e.g., transient fluctuations in power triggered by yarn breakage during the stretching phase, and gradual changes in power during the changeover and reset phases), and these features are dependent

on the bi-directional information of the neighboring time steps (e.g., the relationship between "current peak power" and the "previous moment power"). "previous power baseline" and "subsequent decay trend").

The improved BiLSTM adapts to this requirement by simplifying the gating structure of the traditional LSTM (retaining only one output gate), which reduces the number of parameters and accelerates the training while strengthening the ability to capture bidirectional timing dependencies;

splicing the outputs of the forward and backward LSTMs, so that the features at each time step contain both "historical information" and "future information". " and "future information" (relative to the current step) at each time step, and accurately extracts short-term contextual correlations (e.g., before and after causality of power mutations in the stretching phase).

Although a single BiLSTM can handle short-term dependencies, it cannot model long-range feature correlations (e.g., differences in the overall power trend of different elastic fabrics), and needs to be coupled with other modules.

②Transformer coding layer: bridging the gap between long-range dependence and global information modeling

The electric power sequence not only has short-term fluctuations, but also has long-range dependence across phases (e.g., the correlation between the "peak power in the stretch phase" and the "rate of power decay in the reset phase" of highly elastic fabrics " correlation of highly elastic fabrics, power pattern consistency across multiple experiments for the same type of fabrics), which are beyond the modeling scope of BiLSTM (limited by the "memory decay" of the loop structure).

The Transformer coding layer's self-attention mechanism addresses this issue by globally considering the correlation of any two time steps in the sequence, capturing long-distance dependencies (e.g., the effect of the "power baseline in the permutation phase" on the "peak in the subsequent stretch phase"); and the multi-attention mechanism. ");

Multi-attention mechanism pays attention to features in different subspaces in parallel (e.g., power amplitude differences due to elasticity differences, time point differences in experimental phase transitions) to achieve a refined modeling of global trends. However, Transformer suffers from the defect of "local detail smoothing" (global computation is easy to ignore the short-term drastic fluctuations), and needs to cooperate with the local feature extraction module.

③TCN Network: Optimizing Variable Length Sequence Processing and Local Feature Extraction

The sequence lengths of fabric tearing experiments are variable (e.g., the reset phase of highly elastic fabrics takes longer and the sequence length is larger than that of low-elastic fabrics) and contain a large number of local high-frequency features (e.g., tiny power noise triggered by vibration of the equipment, transient spikes of yarn breakage), which require highly efficient local modeling and variable-length adaptation capabilities. The TCN fills this gap with the following design.

TCN fills this gap by the following design: dilation causal convolution expands the perceptual field by "dilation factor" to capture local multi-scale features (e.g., power correlations at 1-step, 2-step, and 4-step intervals) without increasing the number of parameters, and at the same time ensures that "future prediction is only possible using historical data " (to avoid future information leakage); the residual block structure solves the gradient degradation problem of deep networks by adjusting the dimensionality through 1×1 convolution and suppressing noise through weight normalization, and at the same time adapts to sequences with different lengths (no need to fix the input dimensions) to reduce the complexity of the model;

replaces the Transformer decoder, removes the text vectorization module (Input Embedding), directly

processing the electric power sequence, avoiding redundant computation and improving the efficiency of local feature extraction.

④ Triple synergy: towards full dimensional modeling of "local details-global trend-variable length adaptation"

A single model cannot cover the complexity of electric power sequences:

BiLSTM only: long range dependency modeling is insufficient (e.g., differences in the overall trend of different elastic fabrics cannot be captured); Transformer only: Short-term details are smoothed (e.g. power peaks during stretching phase are weakened) and model complexity is high (self-attentive computational cost grows with the squared length of the sequence); TCN only: bi-directional time-series information is missing (causal convolution can only utilize historical information and does not capture the effect of "future information" on the current step).

After integrating the three: improved BiLSTM provides bidirectional short-term features to address local contextual correlations; Transformer coding layer provides global long-range correlations to address cross-stage trend modeling; TCN optimizes local feature extraction with variable length adaptation to reduce model complexity and suppress noise.

The paper's ablation experiments verify the necessity of this synergy: removing any module (e.g., after removing TCN, the 4-step prediction RMSE of Dataset 2 increases from 0.0931 to 0.2671, an increase of 68.8%) leads to a significant decrease in the prediction accuracy, which proves the theoretical justification of the integration.

In conclusion, the integration of the three models is a targeted design for the characteristics of "short-term fluctuation and long-term dependence, fixed pattern and length variation" of the electric power sequence, which can realize the full-dimensional time series modeling through the complementary functions, and ultimately satisfy the demand for real-time prediction of the fabric tearing experimental process.

3. The average RMSE of multi-step prediction is 0.0881, the average MAE of multi-step prediction is 0.0609, the average MAPE of multi-step prediction is as low as 3.06%, and the average coefficient of determination (R^2) of multi-step prediction is as high as 0.9572. However, for the very important model training time and testing time, the author did not provide them. The author's method is rather complex and requires providing processing time and conducting explanations.

Answer：

In this paper, in the section "Model Training and Parameterization", we have experimentally documented the model's key time performance indicators: 8 hours for offline training and 200 ms for online incremental learning. This performance is due to the lightweight design of the model: the improved BiLSTM simplifies the gating structure, the TCN controls the parameter size by expanding the convolution, and the Transformer retains only the encoder module, which works in concert to keep the complexity of the model while avoiding an excessive increase in time cost. Compared with the comparison models, BLTT-FT has significantly better multi-step prediction accuracy (average RMSE 0.0881), while the training time is still lower than that of hybrid models such as Transformer-LSTM, and the online update speed meets the demand of real-time monitoring (200 ms latency is much smaller than the experimental operation cycle).

4. Many improved versions of LSTM were not discussed by the authors. For example, the LSTM-Reduction method, the CNN-EFC-BiLSTM method.

Answer：

To address the lack of discussion on the improved version of LSTM, the following explanations can be made in the light of the scenario characteristics and model design goals of this study:

①Targeted direction of improvement: focusing on the timing characteristics and real-time requirements of fabric tearing experiments

The improved BiLSTM in this study (simplified gating structure, retaining the one output gate) is not a generalized improvement, but rather is designed to meet the core requirements of the fabric tearing experiments:

Short-term dynamics of the electric power sequence (e.g., power mutation in the stretching phase) rely on bidirectional timing correlations, but do not require overly complex gating mechanisms (traditional LSTM with three gates will introduce redundant parameters). In the experiment, the short-term dynamics of the electric power sequence (e.g., the power mutation during the stretching phase) relies on bi-directional timing correlations, but does not require an overly complex gating mechanism (the 3 gates of the traditional LSTM introduce redundant parameters); the online prediction needs to support fast and incremental learning (a single update takes 200 ms), and the simplified gating reduces the number of parameters (by 40% compared to that of the traditional BiLSTM), which can directly improve the training and inference speeds. speed.

The core goal of this improvement is to balance accuracy and efficiency, which is strongly bound to the scenario of "real-time monitoring + dynamic updating" in fabric tearing experiments.

②Reasons for not discussing other improved versions: differences in scenario suitability

LSTM-Reduction, CNN-EFC-BiLSTM, and other methods mentioned by the reviewers have significant differences in their design goals and the scenarios of this study:

LSTM-Reduction: focusing on the reduction of model parameters through pruning or quantization, which is used in embedded devices with limited resources, but may lose the ability of temporal feature extraction, which is not the same as that of this study. LSTM-Reduction: focuses on reducing the model parameters by pruning or quantization, which is mostly used in embedded devices with limited resources, but it may lose the ability of time-series feature extraction, which is in conflict with the requirement of "preserving the details of short-term power fluctuations" in this study; CNN-EFC-BiLSTM: extracts local spatial features by convolution (CNN), and then combines them with the time-series modeling of BiLSTM, which is suitable for spatially correlated data (e.g., data from multi-sensor arrays). However, this study is only based on a single-dimensional electric power sequence (no spatial features), and the introduction of CNN will increase the redundant computation, which is contradictory to the design goal of "reducing the complexity of the model.

③ Experimental validation support: The effectiveness of the improved method has been verified in a scenario.

Comparison experiments in the paper show that after the improved BiLSTM is synergized with other modules (Transformer, TCN): the prediction accuracy is significantly better than that of the traditional BiLSTM (e.g., MAE of 1-step prediction of Dataset 1: 0.0489 vs. 0.1102); the training efficiency meets the real-time demand (8 hours of offline training, 0.1102 of offline training, and 1 hour of offline training). The training efficiency meets the real-time demand (8 hours of offline training, 200 ms of online updating), and the accuracy is not degraded due to the simplified gating.

This shows that the proposed improvement is better than the generalized improvement method in terms of "accuracy-efficiency" balance for the characteristics of fabric tearing experiments, and its reasonableness has been verified by scenario-based experiments.

In summary, this study does not discuss other LSTM improvement versions in depth, not ignoring the

related work, but choosing the targeted improvement direction based on the unique needs of the experimental scenarios (one-dimensional timing, real-time updating, and short-term dynamic dominance), and the effect of the improvement has been verified by the dual verification of prediction accuracy and efficiency. The "related work" can be supplemented to explain the differences in scenario suitability of different improvement methods in order to improve the comprehensiveness of the discussion.

5. For machine learning methods, the setting of parameters is very important. The author needs to provide the parameter configuration scheme, the parameter adjustment scheme, and the parameter scheme of the comparison algorithm in detail.

Answer:

Detailed descriptions are given in Tables 3 and 5.

6. The author needs to make a comparison with the methods of the recently published works.

Answer:

Thank you for your valuable suggestions. Regarding the proposal of "comparing the methods with those of recently published works", after careful consideration, we believe that the current study has already fully validated the effectiveness of the proposed methods through targeted experiments, and that further complementary comparisons with more recent works will have limited support for the core conclusions for the following reasons:

① Specificity of the study scenarios

This study This study focuses on the specific industrial scenario of fabric tearing performance testing, with the core focus on predicting the trend of the experimental process through electric power timing data. Existing comparison experiments (Section 5.2) have selected classical models (LSTM, BiLSTM, Transformer, TCN, etc.) and hybrid models (Transformer-LSTM) in the field of time-series prediction for the specificity of this scenario, and verified the effectiveness of BLTT-FT in the areas of "local bi-directional dependency capturing + global Long-range association modeling + low complexity". The related works published in recent years (e.g., studies focusing on wind power prediction and traffic flow prediction) mostly focus on the data characteristics of different scenarios (e.g., stronger stochasticity, longer sequence lengths), and their method designs differ greatly from the scenario suitability of this study, so it is difficult to reflect the actual value of the model in the fabric experimental scenarios in a direct comparison.

② Adequacy of existing comparisons

The paper has systematically verified the accuracy (average RMSE=0.0881, R^2 =0.9572 for multi-step prediction) and generalization ability of BLTT-FT through multi-step prediction experiments on three types of datasets (high/medium/low elasticity fabrics) and the ablation experiments have made it clear that the synergistic effect of each module. Meanwhile, the "Related Work" section has cited representative studies in the field of time-series prediction in recent years (e.g., improvement of TCN by Adamowski et al. in 2023, and study of the attention mechanism by Shukla et al. in 2024), and elaborated on the correlation and difference of the design ideas between the present study and these works (e.g., the simplification of the modules for high-frequency and low-noise industrial data). low-noise industrial data). The existing content clearly demonstrates the innovation and necessity of the research.

③ Focus of the study

The core contribution of this study is to propose an "Improved BiLSTM+Transformer+TCN" synergistic architecture suitable for fabric tearing experiments, rather than a comprehensive comparison of generalized timing prediction methods. Existing experiments have sufficiently demonstrated the superiority of this architecture in the target scenarios, and additional comparisons with recent works may

distract from the core innovation and make it difficult to provide a rigorous analysis of the differences in methods for different scenarios in a limited space.

In conclusion, we believe that the existing experimental design and discussion are sufficient to support the research conclusions, and additional comparison with recent works will not significantly enhance the scientific validity and persuasiveness of the paper. Thank you for your understanding and correction.

Reviewer #4:

(1) The abbreviations that appear for the first time should be given their corresponding full names, such as TCN, MAE, RMSE, and MAPE in the abstract

Answer：

Thanks for the suggestion, it has been changed in the article.

(2) The full name and abbreviation should not be repeated, for example, N in TCN is Network. So, the TCN network is incorrect.

Answer：

Thanks for the suggestion, it has been changed in the article.

(3) The parameters of the comparison algorithm should also be provided. Detailed structural parameters should be provided.

Answer：

This is explained in detail in the table Table 10. Model Naming.

(4) The introduction related to the experimental samples should be more detailed.

Answer：

We thank the reviewers for their valuable comments. We have added the key characteristics of the samples according to the actual research, which have been reflected in detail in the section of “Experiment and parameter setting”, in order to illustrate the diversity and validity of the samples more clearly, and to ensure the reliability and generalization of the experimental conclusions.

(5) More comparative algorithms should be added.

Answer：

We thank the reviewers for their valuable suggestions. Regarding the proposal of “adding more comparative algorithms”, we carefully reviewed the existing experimental design of the paper, considering that the currently included comparative models (LSTM, BiLSTM, Transformer, TCN, Transformer-LSTM) have already covered the mainstream of time series forecasting. paradigms - including classical recurrent neural networks (LSTM, BiLSTM), self-attention mechanism models (Transformer), temporal convolutional models (TCN), and typical hybrid architectures (Transformer-LSTM), and the proposed BLLSTM is systematically compared with the proposed LSTM through multiple sets of experiments systematically compare the performance differences of the proposed BLTT-FT model under different elastic fabric datasets and different prediction step sizes, which have been able to clearly demonstrate the advantages of BLTT-FT in terms of multistep prediction accuracies (e.g., lower MAE, RMSE, MAPE, and higher R^2) and long-range dependency capturing capabilities.

In addition, the synergistic effect of BLTT-FT modules (Bi-LSTM, Transformer, TCN) is further verified by ablation experiments in the paper, and the comparative analyses with single-module and two-module combination models (e.g., TCN, Bi-LSTM, Trans-TCN, etc.) have sufficiently supported the innovativeness of the model design. Taken together, the existing comparative algorithms have been able to effectively highlight the technical features and performance advantages of the proposed models, and too much

supplementation may lead to redundant experimental results and weaken the focus of the core conclusions. Therefore, we believe that the current comparative experimental setup is sufficient to verify the validity of the study, and we will not add more comparative algorithms for the time being.
We thank the reviewers again for their understanding and suggestions, and we will further optimize the presentation of the experimental results to ensure that the logic of the existing comparison is clearer and the conclusions are more prominent.

Reviewer #6: The manuscript has been well revised and supplemented. There are still some minor issues and suggestions as follows:

1.Add comparative literature (such as comparing with pure Transformer) in the introduction or discussion, and emphasize how the synergy between modules (such as BiLSTM handling bidirectional dependencies, TCN reducing complexity) addresses the shortcomings of existing methods.

Answer：

Thank you for your careful review and valuable suggestions. Regarding the request to “add a comparison with pure Transformer and emphasize the synergy of modules”, we have carefully checked the original text and found that the relevant content has been reflected in the existing chapters, as follows:

① Comparison with pure Transformer

The original text has already discussed the limitations of pure Transformer (e.g., insufficient local bi-directional dependency capture, high model complexity, global information redundancy, etc.) in the "0 In the “Introduction”, the limitations of pure Transformer (e.g., insufficient capture of local bidirectional dependence, high model complexity, global information redundancy, etc.) are discussed, and the shortcomings of Transformer in dealing with high-frequency time-series data of fabric tearing experiments are clarified; in the “5 Experimental Results and Discussion”, BLT is quantified by ablation experiments. In “5 Experimental Results and Discussion”, the performance difference between BLTT-FT and pure Transformer is quantified through ablation experiments: for example, in the 1-step prediction of Dataset1, the MAE of BLTT-FT (0.0489) is 57.9% lower than that of pure Transformer (0.1196), and in the 9-step prediction, the RMSE is 0.1001 lower than that of pure Transformer (0.1696). In the 9-step prediction, the RMSE (0.1001) is 42.1% lower than that of the pure Transformer (0.1695), which directly verifies the complementary effect of the introduction of the improved BiLSTM and TCN to the pure Transformer.

② Module synergy and the solution to the defects of the existing method

The module synergy mechanism has been elaborated in detail in the section “1.2 Network construction of BLTT-FT system” in the original paper “2 Research Methods”:

Improved BiLSTM captures the local forward-backward dependency through the bi-directional units, and compensates the pure Transformer's dependency on pure Transformer by the improved BiLSTM and TCN. Improved BiLSTM captures local backward and forward dependencies through bidirectional units to make up for the lack of sensitivity of pure Transformer to short-range dynamics;

Self-attention mechanism of Transformer coding layer mines cross-cycle global correlations to solve the defect of “local vision” of single recurrent network;

TCN reduces the complexity of the model by expanding causal convolution and residual connection to overcome the problem of high computational cost of pure Transformer. Transformer's high computational cost problem.

The progressive modeling (local details→global association→complexity control) of the three models is also verified in “5 Experimental Results and Discussion”: compared with pure Transformer and single TCN,

BLTT-FT performs better in the balance of accuracy and efficiency, which confirms that the collaborative design is a targeted solution to the shortcomings of the existing methods. This confirms that co-design is a targeted solution to the shortcomings of existing methods.

The above content has formed a complete logical chain of "theoretical analysis - method design - experimental validation", and if we need to further strengthen it, we can add more specific details of cross-model comparison in the discussion section, but the current discussion can support the core conclusions. Thank you for your understanding and recognition.

2. Supplement the cross-validation process and explain the sample selection strategy to ensure the reliability of generalization ability assessment.

Answer:

A detailed description has been given in "3 Data Preparation and Processing and 4 Experiments and Parameter Settings".

3. Add independent ablation of key modules (such as improved BiLSTM vs. standard BiLSTM) to demonstrate the rationality of the simplified design.

Answer:

Thank you for your attention to the details of the model design. Regarding the suggestion of "adding an independent ablation comparison between the improved BiLSTM and the standard BiLSTM", after careful consideration, we believe that the current experimental design can fully justify the simplified BiLSTM, and there is no need to add additional comparisons for the following reasons:

①Targetedness of the Improved Design and Data Suitability

Improvement The simplified single-gate design of BiLSTM is not blindly reducing the structure, but based on the characteristics of the electric power sequence of the fabric tearing experiment: although the sequence is time series data, the bidirectional dependence of the core dynamics (such as the power surge during the stretching phase and the smooth transition during the reset phase) is relatively clear, and the noise is low. The 3-gate structure of the standard BiLSTM (oblivion gate, input gate, output gate) is mainly used for processing high-noise, long-range dependency complex sequences (e.g., natural language, irregular sensor signals), while the regularity of the data in this study makes redundant gating limited to performance enhancement. The soundness of this targeted design has been verified by the excellent performance of the full model on three types of datasets (multi-step prediction average R^2 = 0.9572).

②Existing experiments have indirectly validated the effectiveness of the module

The original ablation experiments (Section 5.4) have confirmed the BiLSTM in capturing bidirectional localized dependencies by comparing the "full model" with the "Trans-TCN model with BiLSTM removed", which has confirmed BiLSTM's The central role of BiLSTM in capturing bi-directional localized dependencies has been demonstrated by comparing the "full model" with the "Trans-TCN model with BiLSTM removed" (e.g., the 1-step MAE of Dataset1 drops from 0.1162 to 0.0489). At the same time, the overall efficiency of the model is balanced with high accuracy, which in itself demonstrates the advantage of simplified BiLSTM in the "performance-complexity" trade-off - the increase in the number of parameters and the training time is not as significant as in this study, if standard BiLSTM is used. If standard BiLSTM is used, the increase in the number of parameters and training time is contrary to the application goal of "real-time prediction of industrial scenarios" in this study, while the existing design has already realized the optimal fit between efficiency and accuracy.

③Integral validation of module synergy

The core innovation of this study lies in the synergistic architecture of "Improved BiLSTM+Transformer+TCN": BiLSTM captures local bidirectional dependencies, Transformer models global

associations, and TCN reduces complexity, and all three are indispensable. Comparing the improved BiLSTM with the standard BiLSTM alone may fragment the synergistic logic between the modules and fail to reflect the actual value of simplified design in the overall model. The generalization ability of the current full model (metric fluctuation < 5% across resilience level fabrics) has sufficiently demonstrated that the adaptability of the simplified BiLSTM with other modules is superior to the redundant structure of the standard BiLSTM.

In conclusion, we believe that the existing experimental design has sufficiently justified the simplified BiLSTM in terms of data characteristics, overall performance, and collaborative logic, and that additional independent ablation comparisons will have limited effect on strengthening the conclusions. Thank you for your understanding and suggestions.

4. Some references are relatively old. It is recommended to supplement papers related to time series prediction in the past two years. The literature review could benefit from citing the following recent works to provide a broader perspective:
https://doi.org/10.1007/s12145-024-01524-y
https://doi.org/10.2166/hydro.2023.172

Answer：

Thank you for your attention to the timeliness of the references. Regarding the suggestion of supplementing the time series prediction papers in the past two years, after careful consideration, we believe that the existing reference configurations have been able to satisfy the demonstration needs of this paper, mainly for the following reasons:

Core supportive role of the existing literature: this paper focuses on the time series prediction of a specific industrial scenario, namely, fabric tearing experiments, and the theoretical foundations and key application frameworks of the core methods (BiLSTM, Transformer, and TCN) have been fully described in the existing references. The theoretical foundation and key application framework of the core methods (BiLSTM, Transformer, TCN) have been fully described in the existing references. Although some of these papers were published 3-5 years ago, they are still recognized as classic works in the field, and their proposed model architectures (e.g., Transformer's self-attention mechanism, TCN's causal convolution design) are the direct theoretical basis of the BLTT-FT model in this paper, and the seminal role of the classic papers is difficult to be replaced by the derivative studies in recent years.

Focus of research innovation: The innovation of this paper lies in the integration and scenario adaptation of classical models (e.g., improving BiLSTM to adapt to the bidirectional local dependence of fabric power sequences, and balancing the accuracy and efficiency through module synergy) rather than proposing a brand-new time-series prediction algorithm. Existing references have clearly presented the strengths and limitations of the underlying models, which are sufficient to support the logic of this paper's argument of “optimizing the model for the scenario”, and further supplementation of the methodology papers in the past two years has a limited role in highlighting the characteristics of this study.

Relevance of domain relevance: Although the recent studies you recommended have new explorations in time-series prediction methods, they mostly focus on generic scenarios (e.g., weather, energy). The core value of this paper is to apply the time series prediction technique to the sub-field of fabric inspection, and the existing references have already included the application research related to industrial experiment monitoring (e.g., equipment condition prediction, sensor data modeling), which is more suitable for the research context of this paper.

In summary, we believe that the existing references can clearly support the theoretical basis and innovative positioning of the study, so we do not add new literature for the time being. Thank you for your

understanding and suggestions.

5. The quality of some figures needs to be improved, such as Fig 1、Fig 4、Fig 8、Fig 9, and etc.

Thank you for your suggestion, optimization of the image quality has been done.
