# Supplementary material for: Trend analysis and prediction of fabric tear performance testing processes based on the BLTT-FT model
Source: PLoS One. 2025 Dec 1;20(12):e0336501. doi: 10.1371/journal.pone.0336501 (PMC12668543; doi:10.1371/journal.pone.0336501)
Supplement: Attachment — Submitted filename: Response_to_Reviewers_auresp_4.pdf [file pone.0336501.s005.pdf]

Journal Requirements:

1.If the reviewer comments include a recommendation to cite specific previously published works, please review and evaluate these publications to determine whether they are relevant and should be cited. There is no requirement to cite these works unless the editor has indicated otherwise.

Answer：

Thank you for your suggestions. I have carefully evaluated the cited work, and it is closely related to the research content of our paper. The reviewers' suggestions have further improved the content of our paper and contributed to enhancing the rigor of our paper.

2.Please review your reference list to ensure that it is complete and correct. If you have cited papers that have been retracted, please include the rationale for doing so in the manuscript text, or remove these references and replace them with relevant current references. Any changes to the reference list should be mentioned in the rebuttal letter that accompanies your revised manuscript. If you need to cite a retracted article, indicate the article's retracted status in the References list and also include a citation and full reference for the retraction notice.

Answer：

Thank you for the reminder. We have conducted a comprehensive check on the recommended list of references and the in-text citations as required, to ensure the completeness and accuracy of the list.

Additional Editor Comments :

1. Improve the resolution and labeling of figures.

Answer：

We have uploaded the graphic files to the PACE digital diagnostic tool for professional verification, ensuring that the graphics resolution meets the standards of your journal.

2.Include a brief discussion on the impact of environmental variables on model performance.

Answer：

Thank you for the reviewers' valuable suggestions. We fully agree with the potential impact of environmental variables on model performance. Now, combining the research background and experimental design, we supplement a brief discussion on the impact of environmental variables as follows:

In the design and data collection stages of the fabric tear performance testing experiment in this study, full consideration has been given to the potential interference of environmental variables (with temperature and relative humidity as the core) on fabric physical properties and equipment operating status. Strict control of the experimental environment has been implemented to reduce their impact on model performance. Specifically, the entire experiment strictly followed the international standard ISO 139-1973 Textiles - Standard atmospheres for conditioning and testing. The laboratory environment was stably controlled within the range of temperature 20.0 ± 2.0 ℃ and relative humidity 65.0 ± 4.0%. All fabric samples were pre-treated in this environment for 24 hours to eliminate changes in fabric physical properties such as elasticity and strength caused by temperature and humidity fluctuations. This ensured that the fluctuation of electrical power parameters during the experiment mainly originated from the mechanical response of the fabric tearing process, rather than environmental interference.

From the perspective of model performance verification results, under the above-mentioned standardized environment, the BLTT-FT model maintained excellent prediction accuracy on high, medium, and low

elasticity fabric datasets, and its cross-dataset generalization ability was stable. This indicates that in laboratory scenarios where environmental variables are controllable, the model can effectively capture the mapping relationship between electrical power sequences and the fabric tearing process.

It should be noted that the core objective of this study is to verify the effectiveness of the "multi-step prediction model based on electrical power sequences" in a standardized experimental environment. Therefore, environmental variables have not been incorporated into the model as input features for the time being. In subsequent studies, we plan to further expand the analysis of the impact of environmental variables. On the one hand, we will collect environmental parameters such as temperature and humidity synchronously through multiple sensors and build an extended dataset to analyze the variation law of electrical power sequences during the fabric tearing process under different environmental conditions (such as high temperature and high humidity, low temperature and low humidity). On the other hand, by adding environmental variable features to the model input layer, we will verify their supplementary role in improving prediction accuracy. Especially for industrial on-site scenarios with large environmental fluctuations, we will optimize the model's anti-interference ability to make the research results more in line with the needs of intelligent quality control in actual production.

The above supplementary content has been integrated into "3 Data Preparation and Processing". At the same time, the subsequent research direction of environmental variable optimization has been further clarified in the section "6 Limitations and Prospects" to ensure the completeness and logic of the discussion.

3. Update the references to include more recent works from 2024 – 2025, especially those focusing on real-time monitoring in textile processes.

Answer:

Thank you for the reviewers' constructive suggestions. We fully agree with the importance of supplementing recent relevant literature to improve the research background and reflect the timeliness of the study. We have systematically searched and screened the latest research results focusing on real-time monitoring of textile processes from 2024 to 2025, supplemented and updated the reference list, and this information is reflected in "1.1 The Application of Artificial Intelligence in Fabric Performance Prediction".

4. Perform a final proofread to correct minor language errors.

Answer:

Revisions have been made. Thank you for your suggestions.

Reviewer #4:

The ? in [] as [?] for cite reference should be avoid. The ? in [] as [?] for cite reference should be avoid. The ? in [] as [?] for cite reference should be avoid. The ? in [] as [?] for cite reference should be avoid. The ? in [] as [?] for cite reference should be avoid. The ? in [] as [?] for cite reference should be avoid.

Answer:

Revisions have been made. Thank you for your suggestions.
